# Identification of novel therapeutic targets for blocking acantholysis in pemphigus

**DOI:** 10.1111/bph.15233

**Published:** 2020-09-21

**Authors:** Imke A.K. Burmester, Sarah Flaswinkel, Clara‐Sophie Thies, Anika Kasprick, Mayumi Kamaguchi, Valéria Bumiller‐Bini, Shirin Emtenani, Nick Feldmann, Khalaf Kridin, Enno Schmidt, Nina van Beek, Detlef Zillikens, Christoph M. Hammers, Jennifer E. Hundt, Ralf J. Ludwig

**Affiliations:** ^1^ Lübeck Institute of Experimental Dermatology University of Lübeck Lübeck Germany; ^2^ Department of Dermatology University of Lübeck Lübeck Germany; ^3^ Center for Research on Inflammation of the Skin University of Lübeck Lübeck Germany

**Keywords:** autoimmunity, cell signaling, model system, pemphigus, skin

## Abstract

**Background and Purpose:**

Pemphigus is caused by autoantibodies against desmoglein (Dsg) 1, Dsg3, and/or non‐Dsg antigens. Pemphigus vulgaris (PV) is the most common manifestation of pemphigus, with painful erosions on mucous membranes. In most cases, blistering also occurs on the skin, leading to areas of extensive denudation. Despite improvements in pemphigus treatment, time to achieve remission is long, severe adverse events are frequent and 20% of patients do not respond adequately. Current clinical developments focus exclusively on modulating B cell function or autoantibody half‐life. However, topical modulation of PV autoantibody‐induced blistering is an attractive target because it could promptly relieve symptoms.

**Experimental Approach:**

To address this issue, we performed an unbiased screening in a complex biological system using 141 low MW inhibitors from a chemical library. Specifically, we evaluated PV IgG‐induced Dsg3 internalization in HaCaT keratinocytes. Validation of the 20 identified compounds was performed using keratinocyte fragmentation assays, as well as a human skin organ culture (HSOC) model.

**key Results:**

Overall, this approach led to the identification of four molecules involved in PV IgG‐induced skin pathology: MEK1, TrkA, PI3Kα, and VEGFR2.

**Conclusion and Implications:**

This unbiased screening revealed novel mechanisms by which PV autoantibodies induce blistering in keratinocytes and identified new treatment targets for this severe and potentially life‐threatening skin disease.

**Abbreviations**
Dsg: desmoglein
HSOC: human skin organ culture
NC: negative control
PF: pemphigus foliaceus
PV: pemphigus vulgaris

**What is already known**

- Binding of pemphigus autoantibodies induces signalling in keratinocytes.
- Pemphigus treatment aims to stop autoantibody production and/or lower autoantibody concentrations.

**What this study adds**

- Identification of MEK1, TrkA, PI3Kα, and VEGFR2 as therapeutic targets for topical treatment of pemphigus

**What is the clinical significance**

- Identification of topical inhibition of signalling kinases as a novel therapeutic principle in pemphigus

## 1 INTRODUCTION

Pemphigus is a group of prototypical, organ‐specific autoimmune diseases characterized and caused by autoantibodies targeting desmosomal adhesion molecules (Hammers & Stanley, 2016; Kasperkiewicz et al., 2017; Ludwig et al., 2017; Pollmann, Schmidt, Eming, & Hertl, 2018). The clinical disease manifestation largely depends on the autoantibody specificities. In the presence of anti‐desmoglein (Dsg) 1 autoantibodies, intraepidermal blistering occurs solely on the skin, causing pemphigus foliaceus (PF). If Dsg3 autoantibodies are present, lesions are restricted to mucosal sites, causing mucosal pemphigus vulgaris (PV). If both Dsg1 and Dsg3 autoantibodies are present, the skin and mucosal sites are affected, which is termed mucocutaneous PV. The particular disease manifestation on the skin and/or mucous membranes is thought to be caused by the differential expression patterns of Dsg1 and Dsg3 in these tissues (Mahoney et al., 1999; Payne, Hanakawa, Amagai, & Stanley, 2004). In addition to Dsg1 and/or Dsg3 autoantibodies, several other autoantibodies have been detected in pemphigus patients and functionally validated, for example desmocollin 3 (Chernyavsky, Amber, Agnoletti, Wang, & Grando, 2019; Lotti et al., 2019; Sinha & Sajda, 2018). These non‐Dsg1/3 autoantibodies are, however, less frequent compared to Dsg1/3 autoantibodies. Antigens of potentially pathogenic non‐Dsg autoantibodies include desmocollin 3, M_3_ muscarinic receptors and the secretory pathway Ca^2+^/Mn^2+^‐ATPase isoform 1 (Amber, Valdebran, & Grando, 2018; Chernyavsky et al., 2019; Sinha & Sajda, 2018).

Currently, the treatment of pemphigus is based on non‐specific, long‐term systemic immunosuppression, including high doses of corticosteroids. Despite recent significant improvements in pemphigus treatment (Joly et al., 2017; Schmidt et al., 2017), the rate of adverse events in patients is still high. Hence, novel treatment options for pemphigus are still needed. Based on the current understanding of pemphigus pathogenesis, three different treatment targets have emerged: (a) elimination of autoreactive B cells; (b) modulation of autoantibody (IgG) half‐life; and (c) inhibition of the blister‐inducing activity of autoantibodies. Currently, the elimination of autoreactive B cells, as well as all other B cells, is achieved by the anti‐CD20 antibody rituximab (Joly et al., 2017). Furthermore, low MW inhibitors of Bruton tyrosine kinase (BTK), which is essential for B cell activation and maturation (Corneth, Klein Wolterink, & Hendriks, 2016), have been successfully evaluated in a phase I clinical trial in pemphigus patients (Murrell, 2018). In pemphigus, blockade of the B cell activating factor of the tumour necrosis factor family receptor (BAFF receptor) and infusion of ex vivo expanded autologous regulatory T cells are also currently being evaluated in clinical trials (Izumi, Bieber, & Ludwig, 2019). In preclinical settings, autoreactive B cells were selectively depleted by chimeric autoantigen receptor T cells (Ellebrecht et al., 2016). The half‐life of the circulating IgG antibodies directed against Dsg1/3 or the pathogenic non‐Dsg autoantigens in pemphigus can be reduced by blockade of the neonatal Fc receptor, and phase I studies have shown promising results in pemphigus (Lee et al., 2018). In line with these developments, adjuvant immunoadsorption (IA) has been successfully applied in severe and/or refractory cases, and Dsg1/3‐specific adsorbers are currently being developed (Hofrichter et al., 2018; Langenhan et al., 2014). However, B cell targeting treatments need time until the clinical manifestation improves (Joly et al., 2017). Hence, agents that improve symptoms in the acute phase of the disease would fill a so far unmet medical need.

Finally, pemphigus may also be treated by inhibiting the blister‐inducing activity of the autoantibodies. There is still ongoing debate concerning which precise molecular mechanisms contribute to blister formation induced by autoantibodies (Spindler & Waschke, 2018). However, multiple lines of evidence point towards a pemphigus autoantibody‐induced aberrant signalling in keratinocytes after antibody binding to the target antigen(s) (Pollmann et al., 2018), and this signalling involves p38 MAPK, PKC, cJNK, RhoA, and several caspases, as well as signalling downstream of the epidermal growth factor receptor (Berkowitz et al., 2005; Cirillo, Lanza, & Prime, 2010; Grando, 2012; Hariton et al., 2017; Sayar et al., 2014). Among these signalling events, p38 MAPK is best characterized. Specifically, pharmacological p38 MAPK blockade prevents blister formation in an antibody transfer‐based model of pemphigus in neonatal mice (Berkowitz et al., 2006; Berkowitz, Chua, Liu, Diaz, & Rubenstein, 2008). Thus far, these insights have not been translated to patient care, and all current strategies for developing new treatments for pemphigus focus exclusively on the removal of autoreactive B cells or autoantibodies (Lee et al., 2018).

Furthermore, and in contrast to other autoimmune skin blistering diseases (Joly et al., 2002), topical treatment is not considered helpful in PV patients (Eming et al., 2015). Nonetheless, local blockade of signalling events would in principle be a great addition to the treatment of pemphigus because of the relatively long time needed to induce a clinical response with B cell depletion strategies. In addition, cutaneous application of low MW inhibitory molecules may be more tolerable than systemic administration. To address this issue, we first obtained a comprehensive and functional overview of signalling events driving pemphigus pathogenesis in keratinocytes after incubation with pemphigus IgG. Subsequently, based on this understanding, we evaluated the potential therapeutic use of the identified therapeutic targets.

## 2 METHODS

### 2.1 Experiments with human biomaterial

All experiments with human samples were reviewed by the Ethical Committee of the Medical Faculty of the University of Lübeck (reference numbers: 12‐178 and 06‐109) and were performed in accordance with the Declaration of Helsinki. All patients gave their written informed consent for the use of the serum or skin for research purposes.

Sera (*n* = 10) or immunadsorption materials (*n* = 2) from PV patients were taken from the remaining volume after routine diagnosis or from the discarded immunadsorption material. IgG from patient serum was isolated using protein G (cat. # 19459, Sigma‐Aldrich, Taufkirchen, Germany), following established protocols (Kasprick, Bieber, & Ludwig, 2019). Each isolated IgG from PV patient serum or immunadsorption material was tested to induce Dsg3 internalization (see below) before use in our work. Human skin was obtained from elective plastic surgery. Diagnosis of PV was based on the clinical presentation and the presence of anti‐Dsg1 and anti‐Dsg3 autoantibodies by ELISA (Euroimmun AG, Lübeck, Germany) (Schmidt et al., 2015). The IgG concentration of each serum/IA material was determined by NanoDrop.

### 2.2 Selleckchem Target Selective Inhibitory Library

The Target Selective Inhibitory Library (Selleckchem, Munich, Germany) purchased at the time of the screening contains 141 different substances that inhibit signalling pathways (Table S1). All of the substances were diluted in 100% DMSO (Sigma‐Aldrich) and stored at −80°C until use.

### 2.3 Experimental design and group sizes

Experimental design and analysis and their reporting follow the relevant editorial in the *BJP* (Curtis et al., 2018). For an initial screening, the effects of compounds from the Target Selective Inhibitory Library on Dsg3 internalization were determined. To balance between target discovery and experimental workload, we decided to perform the screening with an *n* of 2, which is similar (or even higher) to other drug screenings (Zhang, Wu, & Sills, 2005). The keratinocyte dissociation assay with HaCaT (CVCL_0038) cells was performed with nine independent replicates per group. If less than nine replicates were included (never less than seven), this was due to technical or procedural failures, that is, number of cells not sufficient for all groups. For validation of results obtained in the keratinocyte dissociation assay in HaCaT cells, as well as excluding the possibility that the effects observed were due to any mutations specific for HaCaT cells, we repeated these experiments using NHEK cells from single adult donors (PromoCell, Heidelberg, Germany), which are primary keratinocytes derived from different individual donors. Experiments with NHEK were performed with nine replicates per group. If less than nine replicates were included (never less than eight), this was due to technical or procedural failures. Each of the experiments using PV IgG was performed with at least three different PV IgG preparations to introduce variability. For each experiment, 3 of the 10 PV IgG preparations were randomly selected. Once a given PV IgG preparation was selected, it was again used for randomization if all other preparations had been used. Next, the human skin organ culture (HSOC) model was used to validate these findings in an ex vivo model of pemphigus. In order to have a sufficient number of controls, negative and positive control skin specimens were always performed in duplicates, and data from both (if applicable) were included. In each experiment, inhibitors were investigated in one replicate per dose. Hence, the *n* for the controls is 16 (negative control [NC]) and 19 (positive control), while the *n* for the inhibitors ranges from 7 to 11. For experiments using selumetinib in DAC base cream, six experiments per condition were performed. For technical reasons, one replicate of the “selumetinib intradermal” group was excluded, which explains the unequal sample size in these experiments. Non‐inclusion of experiments was due to technical or procedural failures, that is, false positive/negative controls. Finally, a literature search was performed for the compounds successfully validated in the HSOC model.

### 2.4 Culture of HaCaT cells

HaCaT cells were cultured in T‐175 cm^2^ cell culture flasks at 37°C and 5% CO_2_ with the cell culture medium Keratinocyte Growth Medium 2 (KGM2; PromoCell) supplemented with Supplement Mix (PromoCell), 0.06 mM CaCl_2_ (PromoCell), and 1% penicillin/streptomycin (PAN Biotech, Aidenbach, Germany). For passaging HaCaT cells, the culture medium was discarded, and the cells were detached from the flask with prewarmed trypsin/EDTA (0.05%/0.02%; PromoCell) at 37°C. The trypsin activity was stopped with FBS (Bio&Sell, Feucht, Germany), and detached cells were collected in a tube and washed twice with prewarmed KGM2. After discarding the supernatant, cells were cultured with KGM2 and seeded in 8‐well chambers, with each well containing a concentration of 5 × 10^5^ cells·ml^−1^ in preparation for the Dsg3 internalization assay.

### 2.5 Dsg internalization assay

All of the following steps were performed within 8‐well chambered cell culture slides (Fisher Scientific, Schwerte, Germany) that were attached to an object slide. The keratinocytes (HaCaT) were cultured until they reached ~95% confluence in KGM2 with a concentration of 0.6 mM Ca^2+^. At this point, the Ca^2+^ concentration was increased to 1.5 mM to induce the formation of cell contacts and the differentiation of the keratinocytes. After 24 h, fresh KGM2 and a substance from the Target Selective Inhibitory Library at a concentration of either 0.1, 1, or 10 μM, diluted in 0.1% DMSO and Dulbecco's PBS (DPBS, Gibco), were added to each well of the 8‐well chamber, according to a predefined pipetting order, which was maintained throughout the experiment. After 2 h of preincubation with the substances, the medium was changed again, and the keratinocytes were exposed to KGM2 with PV IgG from one patient, with an IgG concentration of 160 μg·ml^−1^. Additionally, dilution of the substances in DPBS was performed again and added to the culture wells, and the samples were incubated for another 24 h at 37°C. The supernatant of each well was removed after 24 h, and the wells were washed carefully 3 times with 500 μl of DPBS. For fixation, the cells were incubated with formaldehyde for 10 min at room temperature (RT). The keratinocyte layer was blocked after washing with a blocking buffer containing 10% goat serum, 1% BSA (Roth, Karlsruhe, Germany), and 0.05% sodium azide (Sigma‐Aldrich, Hamburg, Germany) for 20 min at room temperature. The supernatant was removed again, and the keratinocytes were prepared for staining with an anti‐Dsg3 antibody (mouse, monoclonal [6G2C11], IgG1; Acris Antibodies, Herford, Germany) diluted 1:100 in DPBS for 1 h. The washing step with DPBS was repeated 3 times to stain the keratinocytes with a Cy3‐conjugated anti‐mouse secondary antibody (goat, polyclonal; Jackson ImmunoResearch, West Grove, PA, USA) diluted 1:100 in DPBS for 1 h in the dark at room temperature. Following three washing steps with DPBS, the cells were fixed with 100% ethanol for 10 min in the dark. The 8‐well chambers were carefully removed. The object slide was air‐dried briefly, then mounted with 1 μl of Mowiol (Sigma‐Aldrich), and covered with cover glass. The experiment was performed at 5 different time points using different passages of HaCaT cells. All of the stained slides were stored at 4°C in the dark until examination 2–3 days later under a fluorescence microscope (Keyence, Neu‐Isenburg, Germany). Images at 100× magnification were photographed for later analysis.

### 2.6 Evaluation of Dsg internalization using ImageJ

The slides were examined, and photos were taken from each chamber. The settings for each photo were identical for each series of experiments. The photos were taken from five randomly chosen fields of view on each slide. The photos were taken from spots that were not located close to the edges of the field to prevent high keratinocyte density resulting in higher fluorescence intensity (FI). For each experimental series that usually consisted of ~3–4 slides with eight chamber fields, one field in which only the PV IgG was added served as a positive control, and one field in which normal human IgG (NH IgG) was added served as a NC. To quantify total Dsg3 internalization, the FI of each photo was measured with ImageJ (RRID:SCR_001935, version 1.48, https://imagej.nih.gov/ij/). With the mean FI of all five photos, the mean intensity for one chamber field was calculated. This calculated mean for each inhibitor from the Target Selective Inhibitory Library (cFI) was taken in proportion to the NC, which was set at 100%. Next, the difference in FI between the positive (pcFI) and NCs (set at 100) was calculated (ΔFI). If cFI/ (pcFI + ΔFI/2) was 0.95 or higher, the compound was considered a potential inhibitor of PV IgG‐induced Dsg3 internalization. As the read‐out for Dsg3 internalization was an objective measurement, the investigator performing the analysis was not blinded.

### 2.7 Assessment of compound toxicity

For in vitro validation, toxicity of the eight compounds shown in Figure 2 was evaluated using the MTT Assay Kit from Abcam (cat. # ab211091, Cambridge, UK) following the manufacturer's instructions. For each experiment, cells were incubated with the indicated drugs for 24 h. Experiments were performed in three biological replicates with two technical replicates per experiment whereby the average of the two technical replicates was used as the data point (*n* = 3 per group). We used this exploratory screening of potential toxicity to exclude any major effects.

### 2.8 Culture of normal human epidermal keratinocytes

Analogous to HaCaT cells, normal human epidermal keratinocytes (NHEKs) were cultured in 175 cm^2^ cell culture flasks at 37°C and 5% CO_2_ with the cell culture medium KGM2 supplemented with Supplement Mix (PromoCell) and 0.06 mM CaCl_2_. For passaging NHEKs, the supernatant was discarded, and the cells were washed twice with prewarmed DPBS (Gibco) before adding prewarmed 0.05% trypsin/0.02% EDTA (PromoCell) to detach the cells at 37°C. Trypsin activity was reduced by adding DPBS; afterwards, the solution containing the cells was transferred immediately to a tube and washed twice by centrifugation (3 min, 220 x *g*, room temperature ) with prewarmed KGM2. Subsequently, the supernatant was discarded, and the cells were cultured with KGM2 at a density of ~3,500–5,000 cells·cm^−2^.

### 2.9 Keratinocyte dissociation assay

Fifty thousand HaCaT cells or 40,000–60,000 NHEKs were seeded per well on a 12‐well cell culture plate. The cells were cultivated with low Ca^2+^ KGM2 (as described above) until they reached 95–100% confluency. The medium was then changed to high Ca^2+^ KGM2 with 1.5 mM CaCl_2_. After culturing the cells for 22 h, the compounds were added (at a 0.1, 1, or 10 μM concentration) in 0.1% DMSO/high Ca^2+^ medium, according to a predefined pipetting order, which was maintained throughout the experiment. After 2 h of incubation at 37°C and 5% CO_2_, the supernatant was discarded, and the cells were incubated with immunoapharesis material from a PV patient (2 mg·ml^−1^) in the presence of the inhibitors (same concentrations). For each 12‐well plate, patient material in 0.1% DMSO served as a positive control, and either human IgG (2 mg·ml^−1^ in high Ca^2+^ medium) or high Ca^2+^ medium was prepared as an NC. In experiments using HaCaT cells, immunoapharesis material from different patients was used. In experiments, using NHEKs, each experiment was performed using a unique combination of either patient material and different NHEK cells. The plates were incubated again for 24 h at 37°C in 5% CO_2_. Subsequently, the supernatant was discarded, and the cells were detached as a monolayer by incubating the wells with a 1.25 U·per well dispase–HBSS solution (dispase from Stemcell Technologies Inc., Vancouver, British Columbia, Canada; HBSS from Gibco) for 15–30 min. Afterwards, the cells were washed twice with prewarmed HBSS, 1 ml of HBSS per well was added, and the cell monolayers were then exposed to mechanical stress by pipetting up and down 5 times using a 1‐ml pipette. The tips were coated in 1% BSA for 15 min to prevent the cells from sticking to the pipette tip. Finally, the cell fragments were fixed by adding 4% Roti‐Histofix (Roth) and stained with crystal violet (Sigma). After 1–2 days, photos of the wells were taken with a Gel Documentation System, and cell fragments were counted manually by an investigator unaware of the applied treatments.

### 2.10 Western blotting

HaCaT cells were seeded in 6‐well plates and cultured using KGM2 medium (see above). For the experiment, the medium was changed to KGM2 containing 1.5 mM CaCl_2_ and cultured for 24 h. Next, inhibitors at 1 μM or solvent was added for 2 h. Thereafter, the medium was changed again, and cells were exposed to PV IgG or NH IgG for 24 h. Next, cells were lysed with 1% Triton X‐100 in PBS containing a protease inhibitor cocktail (Thermo Fisher Scientific, Waltham, MA, #1862209), followed by centrifugation at 5,000 x *g* for 20 min. The supernatant was used as the cytosol/membrane fraction. Triton X‐100‐insoluble pellets were solubilized in 2% SDS sample buffer and stored as the cytoskeleton fraction, containing desmosomal components. For the immunoblotting, the samples were applied to the 8% SDS gels and SDS‐PAGE performed. The gels were transferred to a nitrocellulose membrane (Bio‐Rad Laboratories, Hercules, CA, #162‐0146) with transfer buffer (25 mM Tris, 190 mM glycine, and 20% methanol). After blocking for 1 h at RT in 5% skimmed milk (Carl Roth, Karlsruhe, Germany, #68514‐61‐4) in TBS/T, the membranes were incubated with 1:100 diluted anti‐human Dsg3 antibody (Acris, Herford, Germany, #SM2037PS) and 1:1000 diluted anti‐Plakoglobin antibody (Biocompare, San Francisco, CA, #MA1012) with 5% skimmed milk in TBS/T overnight at 4°C. HRP‐conjugated or anti‐mouse (1:1000 dilution; R&D systems, Minneapolis, MN #HAF007) or anti‐rabbit IgG (1:1000 dilution; R&D systems, Minneapolis, MN, #HAF008) in TBS/T was reacted for 1 h at RT. Signals were visualized with Clarity Western ECL Substrate (Bio‐Rad Laboratories, Hercules, CA, #1705060S). The immuno‐related procedures used comply with the recommendations made by the *British Journal of Pharmacology* (Alexander et al., 2018).

### 2.11 The pemphigus HSOC model

The protocol for the HSOC model has been described in detail elsewhere (Burmester et al., 2019). In brief, human skin was cut into 5 × 5 mm sections and stored in a sterile Petri dish containing William's E medium (Gibco) on ice until further use (the skin was used within 24 h after surgery). Prewarmed defined keratinocyte serum‐free medium (D‐K‐SFM; Gibco) was added to the wells of a Transwell cell culture insert plate (Sigma‐Aldrich) to a height that created an air–liquid interface between the epidermis and the air. The inhibitors were prepared at three different concentrations (0.01, 0.1, or 1.0 mM). As an NC, human IgG or DPBS was used. As a positive control, an antibody phage display‐derived human bispecific monoclonal single‐chain variable fragment (scFv) against Dsg3 and Dsg1 was used (Hammers et al., 2015; Payne et al., 2005). For each HSOC, at least two skin specimens were required as positive control or NC, as well as three skin pieces for testing each compound at different concentrations. First, only the inhibitors were injected into the dermis of the skin pieces (volume: 50 μl) before placing the pieces from each well on a Transwell plate. Positive control and NC were injected with DPBS. The Transwell plate with the prepared skin was placed in a humidified incubator (37°C, 5% CO_2_) for 2 h. Subsequently, the same amount of inhibitor per skin piece was injected together with the scFv. The scFv was injected at a concentration of 3.75 μg·μl^−1^ because previous experiments showed that 22.5 μl of this concentration (diluted in a total volume of 50 μl) induced intraepidermal splitting of at least 85% of the entire epidermal length (not shown). Alternatively, one of the inhibitors was also topically applied in skin specimen injected with the anti‐Dsg1/3 scFv. For this, selumetinib (at 1% concentration) was emulsified in DAC base cream (DAC Basiscreme, Pharmacy of the University Hospital, Schleswig Holstein, Campus Lübeck, Germany). Base cream served as control. This experiment was performed in five independent skin organ cultures. Human IgG or DPBS served as an NC, while scFv alone was injected into the positive control skin piece. Allocation of skin specimen to the different groups was randomly performed. Afterwards, the Transwell plates were maintained in the incubator for 24 h. Next, the skin samples were harvested and cut in halves with a sterile scalpel. One half of the skin was fixed in 4% Histofix solution for subsequent paraffin embedding and haematoxylin–eosin (H&E) staining. The other half was embedded in Tissue‐Tek O.C.T. compound (Sakura Finetek, Staufen im Breisgau, Germany) for direct immunofluorescence staining for the binding of the scFv. For each HSOC specimen, nonoverlapping pictures of one H&E‐stained section were taken over the total length of the skin piece (Keyence). Using ImageJ, the percentage of epidermal split formation was quantified by an investigator unaware of the applied treatments.

### 2.12 scFv production and purification

The HA‐tagged anti‐Dsg1/3 scFv was produced in *Escherichia coli* and purified as detailed elsewhere (Payne et al., 2005).

### 2.13 Direct immunofluorescence staining

To show the binding of the HA‐tagged scFv to the epidermis, cryosections of HSOC skin pieces were stained using a high‐affinity rat anti‐HA‐peroxidase monoclonal antibody (Roche) diluted 1:100 in blocking buffer (0.5 g of BSA [Sigma] diluted in a solution composed of 10× Tris‐buffered saline [Bio‐Rad, München, Germany], 0.05% 2 M CaCl_2_ [Acros Organics, New Jersey, USA], 89.96% distilled water, and 10% Tween‐20 [Euroimmun]) as a secondary antibody and Alexa Fluor 594‐conjugated goat anti‐rat IgG (H + L) (Life Technologies, Carlsbad, USA) diluted 1:200 in blocking buffer as a tertiary antibody. For embedding and cell nucleus staining, stained cryosections were mounted with DAPI Fluoromount‐G (Southern Biotech, Birmingham, USA). As this experiment was used as quality control, no blinding was performed.

### 2.14 Data and statistical analysis

If not otherwise indicated, data are presented as mean ± SEM. For statistical analysis, Prism (Version 8, GraphPad Software, San Diego, USA; RRID:SCR_002798) and SigmaPlot (Version 13, Systat Software GmbH, Erkrath, Germany; RRID:SCR_003210) were used. Tests used for each data set are indicated in the Table and Figure legends. Levene's tests were performed to evaluate homogeneity of variance in each one of the comparisons. When the latter test demonstrated significance, the non‐parametric Kruskal–Wallis H Test was utilized and followed by Dunn's post hoc test as indicated. Two‐tailed *P*‐values < 0.05 were considered as statistically significant. For normally distributed data, one‐way ANOVA with Dunnett's post test was used.

### 2.15 Nomenclature of targets and ligands

Key protein targets and ligands in this article are hyperlinked to corresponding entries in the IUPHAR/BPS Guide to PHARMACOLOGY (http://www.guidetopharmacology.org) and are permanently archived in the Concise Guide to PHARMACOLOGY 2019/20 (Alexander, Christopoulos et al., 2019; Alexander, Fabbro et al., 2019a, b; Alexander, Kelly et al., 2019).

## 3 RESULTS

### 3.1 PV IgG‐induced Dsg3 internalization identifies 20 pemphigus treatment targets

**FIGURE 1:**
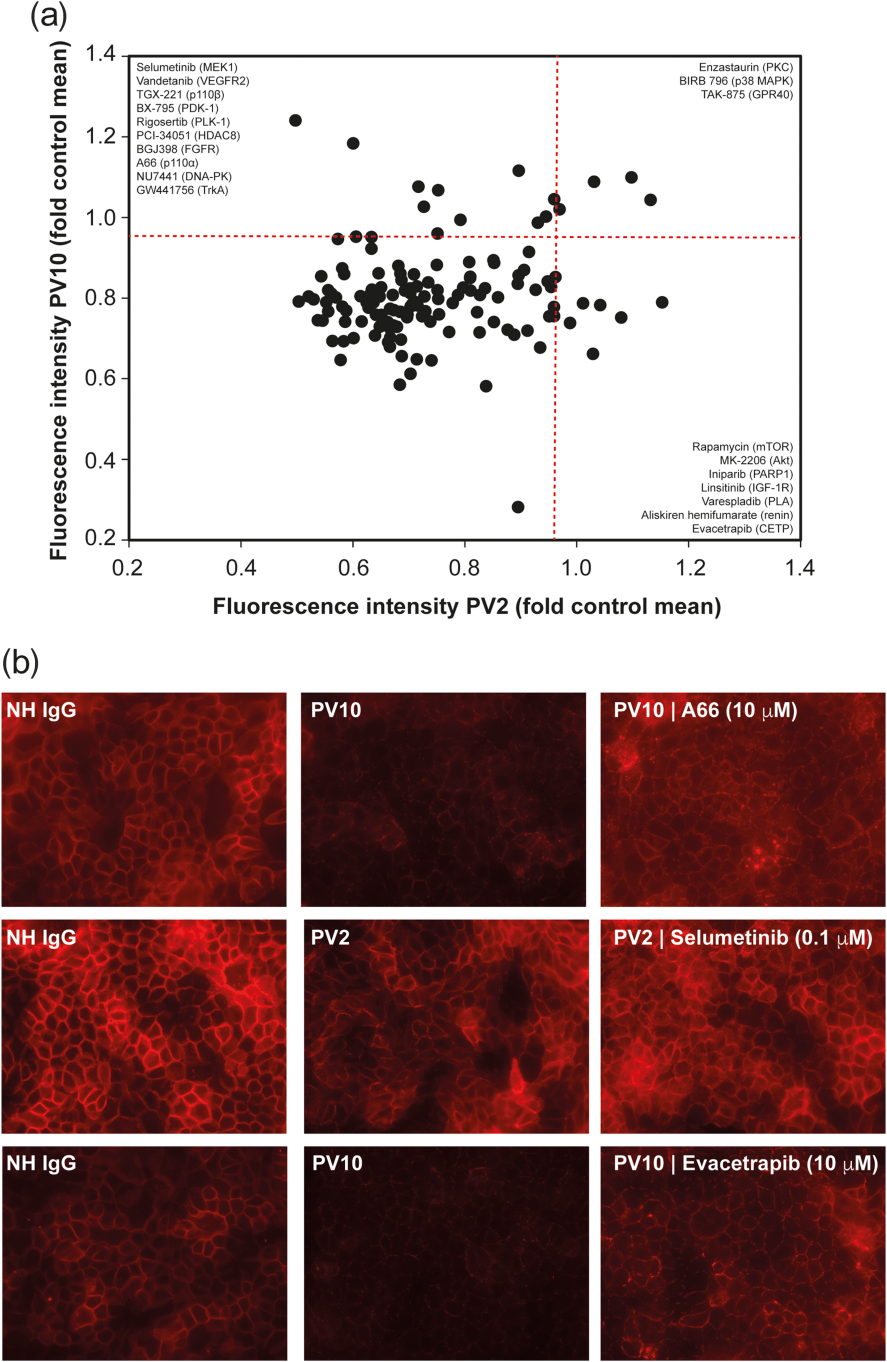
Modulation of PV IgG‐induced Dsg3 internalization in HaCaT cells by target‐selective, low MW inhibitors. (a) HaCaT keratinocytes were incubated with one of the two pemphigus vulgaris (PV) immunadsorption materials, and Dsg3 expression on the cellular surface was determined by immunohistochemistry. A compound was considered to block PV IgG induction if cFI/(pcFI + ΔFI/2) was 0.95 or higher. The red line indicates the predefined cut‐off. All data were normalized to NH IgG. Collectively, 10 compounds inhibited PV10 IgG‐induced Dsg3 internalization (upper left corner), and seven compounds were identified with PV2 and three compounds inhibited Dsg3 internalization for both patients' IgG. (b) Representative images from the experiments shown in panel (a)

In the initial screening, HaCaT cells were incubated with immunadsorption material from two PV patients in the absence or presence of compounds from the Selleckchem Target Selective Inhibitor Library (Table S1). The intraday variability of the FI was low (not shown), whereas the day‐to‐day variability was relatively high. For example, the average SD for NH IgG amounted to 23% and to 28% for PV IgG in the experiments of the PV10 dataset. Of the 141 compounds, 20 prevented pemphigus IgG‐induced Dsg3 internalization from either one or both pemphigus patient IgG preparations by the predefined cut‐off at a concentration of either 0.1 or 10 μM (Figure 1). Inhibitors of p38 MAPK and PKC were among the compounds that prevented PV IgG‐induced Dsg3 internalization. These findings validated the screening approach because these compounds are known to block pathogenicity induced by PV autoantibodies in keratinocytes (Spindler & Waschke, 2018; Walter et al., 2017).

### 3.2 The HaCaT cell dissociation assay validates seven pemphigus treatment targets

**FIGURE 2:**
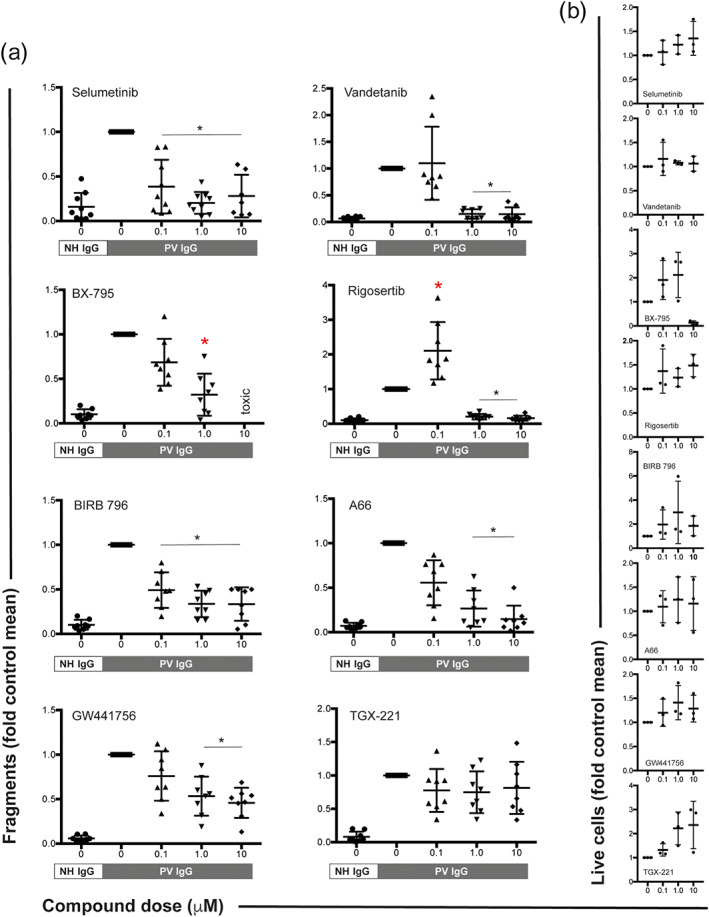
Inhibitors of MEK1, VEGFR2, PDK1, PLK1, p38 MAPK, PI3Kα, and TrkA impair PV IgG‐induced cell fragmentation in HaCaT keratinocytes. (a) HaCaT cell sheaths were exposed to defined mechanical stress in the presence of pemphigus vulgaris (PV) IgG. Normal human (NH) IgG served as a negative control. Inhibitors of MEK1 (selumetinib), VEGFR2 (vandetanib), PDK1 (BX‐795), PLK1 (rigosertib), p38 MAPK (BIRB 796), PI3Kα (A66), and TrkA (GW441756) impaired the PV IgG‐induced fragmentation of the cell sheaths. The remaining 13 compounds (Figure 1) had no effect on cell fragmentation. The PI3Kβ inhibitor TGX‐221 is shown as one example of a compound that had no effect on PV IgG‐induced cell fragmentation. All data were normalized to the fragmentation induced by PV IgG. Data are shown as individual values, with means ± SD from seven to nine independent replicates per group. *P<0.05, significantly different, as indicated, from PV IgG; Kruskal–Wallis H test followed by Dunn's post hoc test. (b) All compounds, with the exception of BX‐795, had no toxic effects at the indicated doses. Data are shown as individual values, with medians ± SD from three replicates per group and is an exploratory screening of the potential toxicity

To validate the 20 potential therapeutic targets identified, we next investigated the effects of these compounds on PV IgG‐induced HaCaT cell dissociation. In this model system, a defined mechanical stress is applied to a cell sheet of HaCaT cells after incubation with PV IgG in the absence or presence of one of the inhibitors at doses of 0.1, 1.0, or 10 μM. Dose‐dependent inhibition of PV IgG‐induced fragmentation was observed for the VEGFR2 inhibitor vandetanib, the PDK1 inhibitor BX‐795, the PLK1 inhibitor rigosertib, the PI3Kα inhibitor A66, and the TrkA inhibitor GW441756. Inhibition independent of the inhibitor dose was observed for the MEK1 inhibitor selumetinib and the p38 MAPK inhibitor BIRB 796 (doramapimod). All other compounds, exemplified by the PI3Kβ inhibitor TGX‐221, had no effect on PV IgG‐induced cell fragmentation (Figure 2a). Of the seven successfully validated compounds, rigosertib was excluded from further validation due to the compound's enhancement of cell fragmentation at 0.1 μM. We further excluded doses of BX‐795 greater than 1 μM due to the potentially toxic effects of that concentration. All other effects were observed at non‐toxic concentrations (Figure 2b).

### 3.3 NHEK cell dissociation validates six pemphigus treatment targets

**FIGURE 3:**
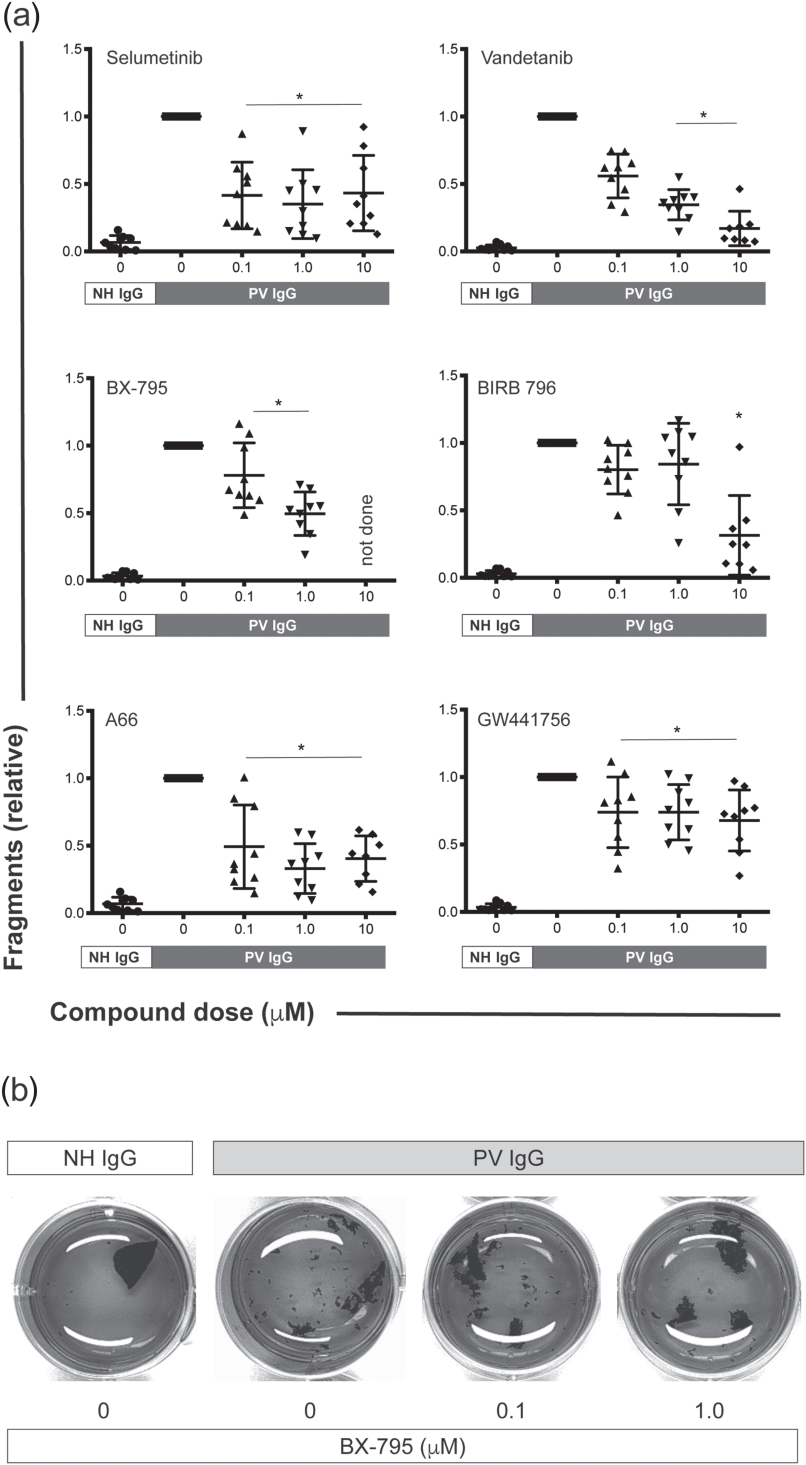
Inhibitors of MEK1, VEGFR2, PDK1, p38 MAPK, PI3Kα, and TrkA impair PV IgG‐induced cell fragmentation in NHEK keratinocytes. (a) NHEK cell sheaths from three donors were exposed to defined mechanical stress in the presence of PV IgG from three different patients. Normal human (NH) IgG served as a negative control. Inhibitors of MEK1 (selumetinib), VEGFR2 (vandetanib), PDK1 (BX‐795), p38 MAPK (BIRB 796), PI3Kα (A66) and TrkA (GW441756) impaired the PV IgG‐induced fragmentation of the cell sheaths. All data were normalized to the fragmentation induced by PV IgG. Data are shown as individual values, with means ± SD from eight to nine independent replicates per group. *P<0.05, significantly different, as indicated, from PV IgG; Kruskal–Wallis H test followed by Dunn's post hoc test. (b) Representative images from one experiment are shown in panel (a). The brightness of all images was increased to the same extent using GIMP (www.gimp.org) to better contrast the sheaths from the medium

To introduce variability, we next repeated the cell dissociation assay using primary keratinocytes originating from three different donors. All six compounds that had been selected in the cell dissociation assay using HaCaT cells also inhibited PV IgG‐induced fragmentation of NHEK cells. Specifically, dose‐dependent inhibition was observed for vandetanib, BX‐795, and BIRB 796. Inhibition independent of the inhibitor dose was detected for selumetinib, A66, and GW441756 (Figure 3).

### 3.4 The HSOC model validates four novel targets for treating pemphigus

**FIGURE 4:**
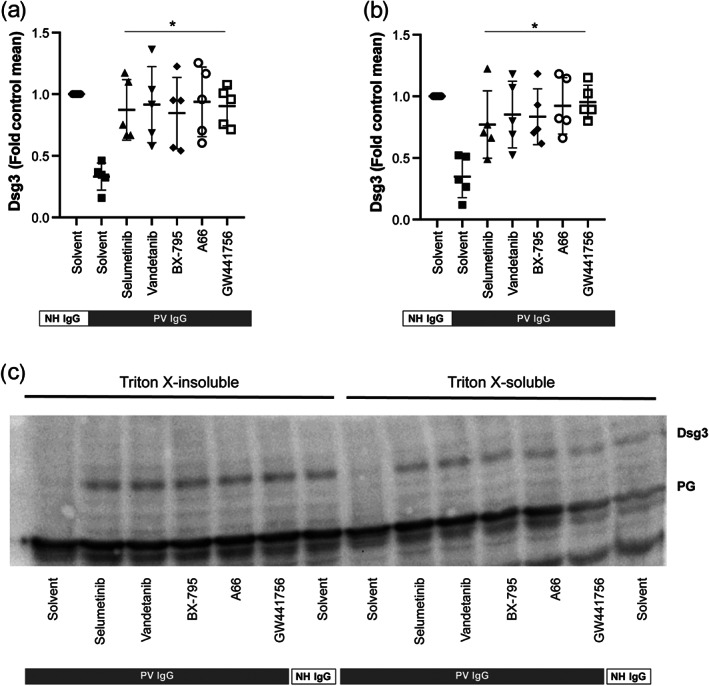
Inhibitors of MEK1, VEGFR2, PDK1, PI3Kα, and TrkA impair PV IgG‐induced internalization of the cytoskeletal‐anchored as well as from non‐cytoskeletal‐anchored Dsg3 pool in HaCaT keratinocytes. PV IgG was added to HaCaT keratinocytes for 24 h and Dsg3 internalization from the cytoskeletal‐anchored and the non‐cytoskeletal‐anchored Dsg3 was assayed using western blotting of Triton‐X lysed cells. (a–b) Inhibitors of MEK1 (selumetinib), VEGFR2 (vandetanib), PDK1 (BX‐795), PI3Kα (A66), and TrkA (GW441756) almost completely inhibited PV IgG‐induced Dsg3 internalization from both Dsg3 pools. Graphs show the relative Dsg3 staining in (a) the Triton X‐insoluble and (b) soluble fraction of HaCaT cells. Data are shown as individual values, with medians ± SD from five replicates per group. *P<0.05, significantly different, as indicated, from PV IgG+Solvent; ANOVA with Dunnett's post test. (c) Representative western blot showing the Triton X‐insoluble and soluble fraction of HaCaT cells 24 h after the addition of PV IgG. Abbreviations used: Dsg3, desmogelein 3; PK, plakoglobin

Next, we evaluated the effects of selumetinib, GW441756, A66, vandetanib, or BX‐795 on PV IgG‐induced Dsg3 internalization using Triton extraction to distinguish between the cytoskeletal‐anchored from the non‐cytoskeletal‐anchored Dsg3 pool. We refrained from testing the p38 MAPK inhibitor BIRB 796 in this assay, because intraepidermal blistering was unaltered in epidermis‐specific p38α‐deficient mice (Mao, Sano, Park, & Payne, 2011), and pemphigus patients treated with p38 MAPK inhibitors experienced no clinical benefit (Sayar et al., 2014). As reported (Yamamoto et al., 2007), we also demonstrate that PV IgG depletes both cytoskeletal‐anchored and non‐cytoskeletal‐anchored Dsg3 from keratinocytes (Figure 4). Treatment with either of the five compounds prevented Dsg3 internalization from both compartments (Figure 4).

**FIGURE 5:**
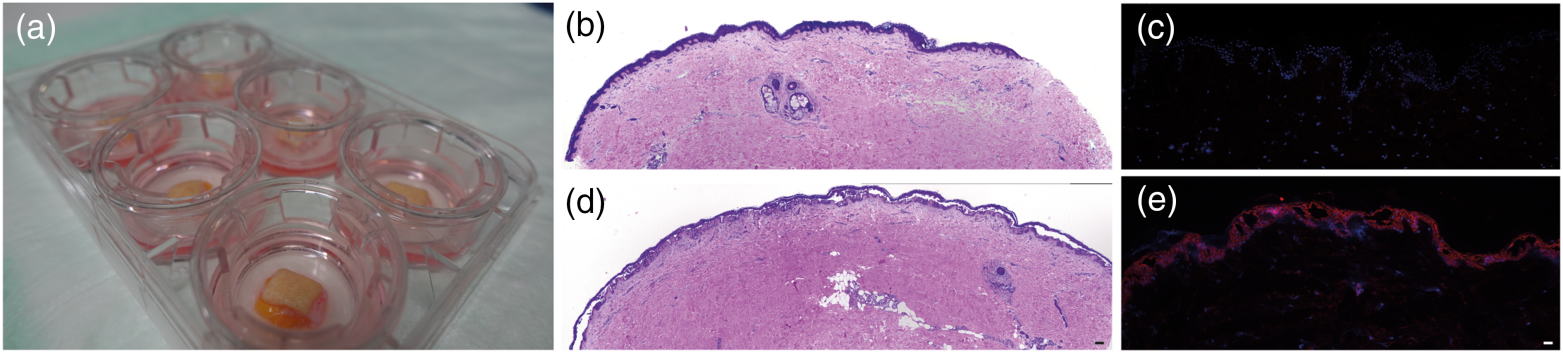
Anti‐Dsg1/3 scFv induces acantholysis in a full thickness human skin organ culture model. (a) Images of six skin specimens in culture. (b) H&E‐stained section from a skin section incubated with normal human (NH) IgG shows no intraepidermal blistering. (c) Anti‐Dsg1/3 scFv‐ (red) and DAPI (blue)‐stained sections from a skin section incubated with NH IgG show no autoantibody binding to keratinocytes. (d) H&E‐stained section from a skin section incubated with pemphigus vulgaris (PV) IgG, with extensive intraepidermal blistering. (e) Anti‐Dsg1/3 scFv‐ (red) and DAPI (blue)‐stained sections from a skin section incubated with anti‐Dsg1/3 scFv show the presence of autoantibodies bound to keratinocytes, as well as intraepidermal blistering. Scale bars correspond to 100 μm

**FIGURE 6:**
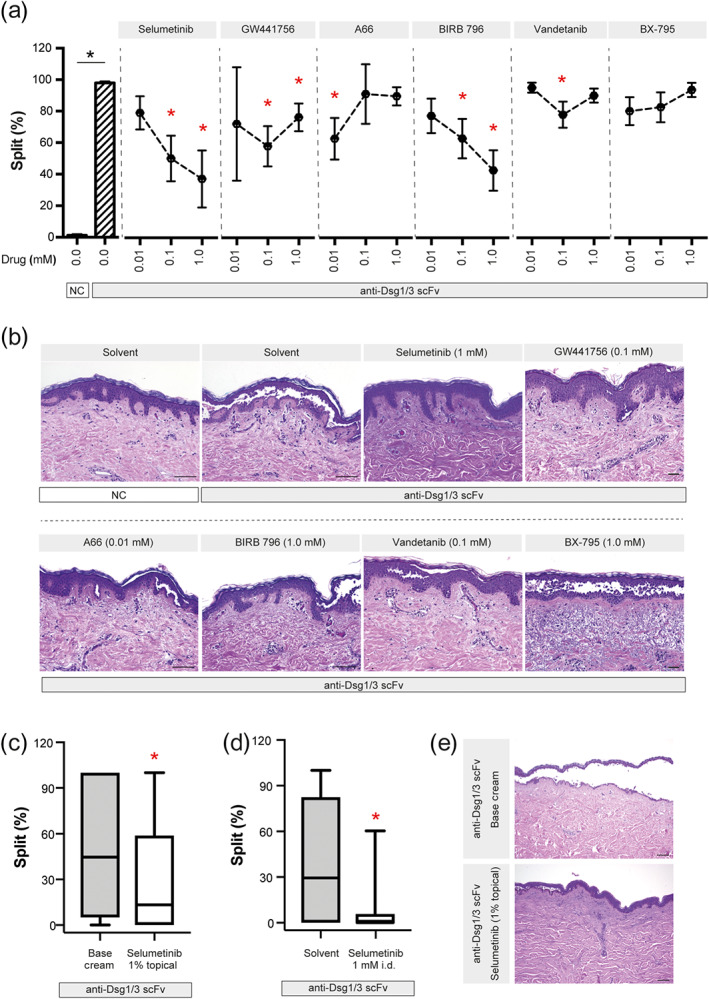
Inhibitors of MEK1, TrkA, PI3Kα, p38 MAPK, and VEGFR2 impair PV IgG‐induced intraepidermal split formation in the human skin organ culture model. (a) The anti‐Dsg1/3 scFv was injected into human skin samples, in the absence or presence of the indicated inhibitors. Data are shown as individual values, with medians ± SD from 8–19 sections per group from three organ cultures. *P<0.05, significantly different from anti‐Dsg1/3 scFv alone; Kruskal–Wallis H test followed by Dunn's post hoc test. (b) Representative H&E‐stained images from the experiments are shown in panel (a). For better contrast, white correction was applied using GIMP (www.gimp.org). Scale bars correspond to 100 μm. (c) The same experimental set‐up as for panel (a), with the exception that selumetinib was emulsified in base cream (1%), which was applied on top of the organ culture. For control, skin specimens were treated with base cream alone. Data are shown as box (first and third quartiles) and whisker (range) plots with medians, and are based on 39–40 sections from four to five organ cultures. ^*^ *P* < 0.05, significantly different from base cream alone; rank‐sum test. (d) The same experimental set‐up as for panel (a), performed in parallel to the experiments from panel (c) as a positive treatment control. Data are shown as box ( first and third quartiles) and whisker ( ) plots with medians and based on 37–44 sections from five to six organ cultures; ^*^ *P* < 0.05, significantly different from solvent alone; rank‐sum test. (e) Representative H&E‐stained images from the experiments are shown in panel (c). For better contrast, white correction was applied using GIMP (www.gimp.org). Scale bars correspond to 100 μm

To further validate our findings in preclinical PV models, we selected a skin organ culture model. Experimental PV can also be induced in mice by transfer of PV IgG into neonatal or adult mice (Anhalt, Labib, Voorhees, Beals, & Diaz, 1982; Schulze et al., 2012). However, we refrained from using this model because the mouse model does not fully reflect the situation encountered in patients (Spindler & Waschke, 2018). To standardize the skin organ culture model, we injected a well‐characterized, pathogenic bispecific recombinant human scFv that binds both Dsg1 and Dsg3 (Hammers et al., 2015; Payne et al., 2005). This scFv consistently leads to the induction of intraepidermal blister formation when injected into normal human skin (Figure 5). In the presence of the anti‐Dsg1/3 scFv, intraepidermal splits were present in the skin specimen, while injection of NH IgG (NC) left the skin intact. If the skin was co‐injected with the MEK1 inhibitor selumetinib, dose‐dependent inhibition of anti‐Dsg1/3 scFv‐induced intraepidermal split formation was observed. Similar observations were made for the p38 MAPK inhibitor BIRB 796. In contrast, inhibition of anti‐Dsg1/3 scFv‐induced skin pathology was independent of the concentration of GW441756 and A66. Despite having demonstrated therapeutic effects in all of the above assays, the PDK1 inhibitor BX‐795 did not modulate anti‐Dsg1/3‐induced acantholysis (Figure 6).

As topical use of these inhibitors may be associated with less adverse events as opposed to systemic application, we evaluated the effects of topical application of one of the inhibitors in the HSOC model. We selected selumetinib because it was among the most potent inhibitors when co‐injected with the anti‐Dsg1/3 scFv (Figure 6a). For this purpose, selumetinib was emulsified in base cream. As a control, the effect of 1 μM selumetinib was investigated in parallel. Topical selumetinib treatment reduced the median split formation from 44.6% to 13.2% (~70% reduction), while co‐injection of the compound reduced median split formation from 63.3% to 6.1% (~90% reduction; Figure 6c–d).

## 4 DISCUSSION

Pemphigus is a potentially life‐threatening disease. Treatment with high doses of corticosteroids in combination with the anti‐CD20 antibody rituximab induces complete remission in the majority of patients (Joly et al., 2017; Kasperkiewicz et al., 2017). However, complete remission is only achieved after 120–180 days of treatment, severe adverse events occur in one third of patients, and 20% of patients do not reach complete remission (Joly et al., 2017). Hence, there is a clear need for the development of novel therapeutic options for pemphigus. Fortunately, several new treatment strategies are under clinical investigation. However, these treatments focus solely on the blockade of autoreactive B cells or enhanced clearance of autoantibodies (Hofrichter et al., 2018; Lee et al., 2018). Previously, application of a tandem peptide directed against Dsg3 was reported to inhibit p38 MAPK activation and keratin filament retraction both in vitro and in the neonatal mouse model of pemphigus. However, this project has not been translated into a clinical application (Heupel et al., 2009; Spindler et al., 2013). Thus, to the best of our knowledge, no treatment targeting autoantibody‐induced intraepidermal blistering is under development.

**FIGURE 7:**
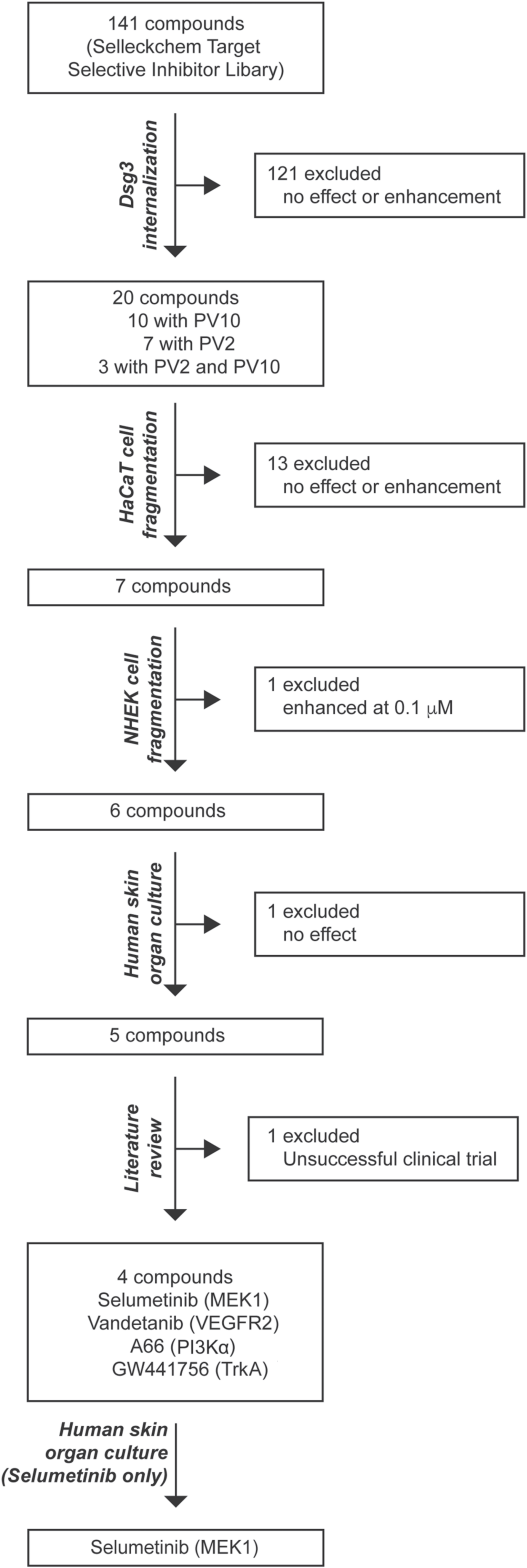
Summary of screening and validation. A total of 141 compounds from the Selleckchem Target Selective Inhibitor Library (Table S1) were used for the initial screening. Of these, 20 compounds were defined as potential inhibitors of PV IgG‐induced pathology in keratinocytes and were used in the first validation experiment. Next, in the HaCaT cell dissociation assay, seven compounds were selected for further validation. In the NHEK cell dissociation assay, 6/6 compounds displayed inhibitory effects on PV IgG‐induced pathology. Ultimately, these remaining compounds were tested in the human skin organ culture model, and 5/6 of the compounds impaired the induction of intra‐epidermal blistering induced by a monoclonal bispecific anti‐Dsg1/3 scFv. Based on the literature (Sayar et al., 2014), we excluded p38 MAPK from the list of potential therapeutic targets. In summary, the screening and subsequent validation identified four novel therapeutic targets for the modulation of PV IgG‐induced intraepidermal blistering, namely, MEK1, TrkA, PI3Kα, and VEGFR2

**FIGURE 8:**
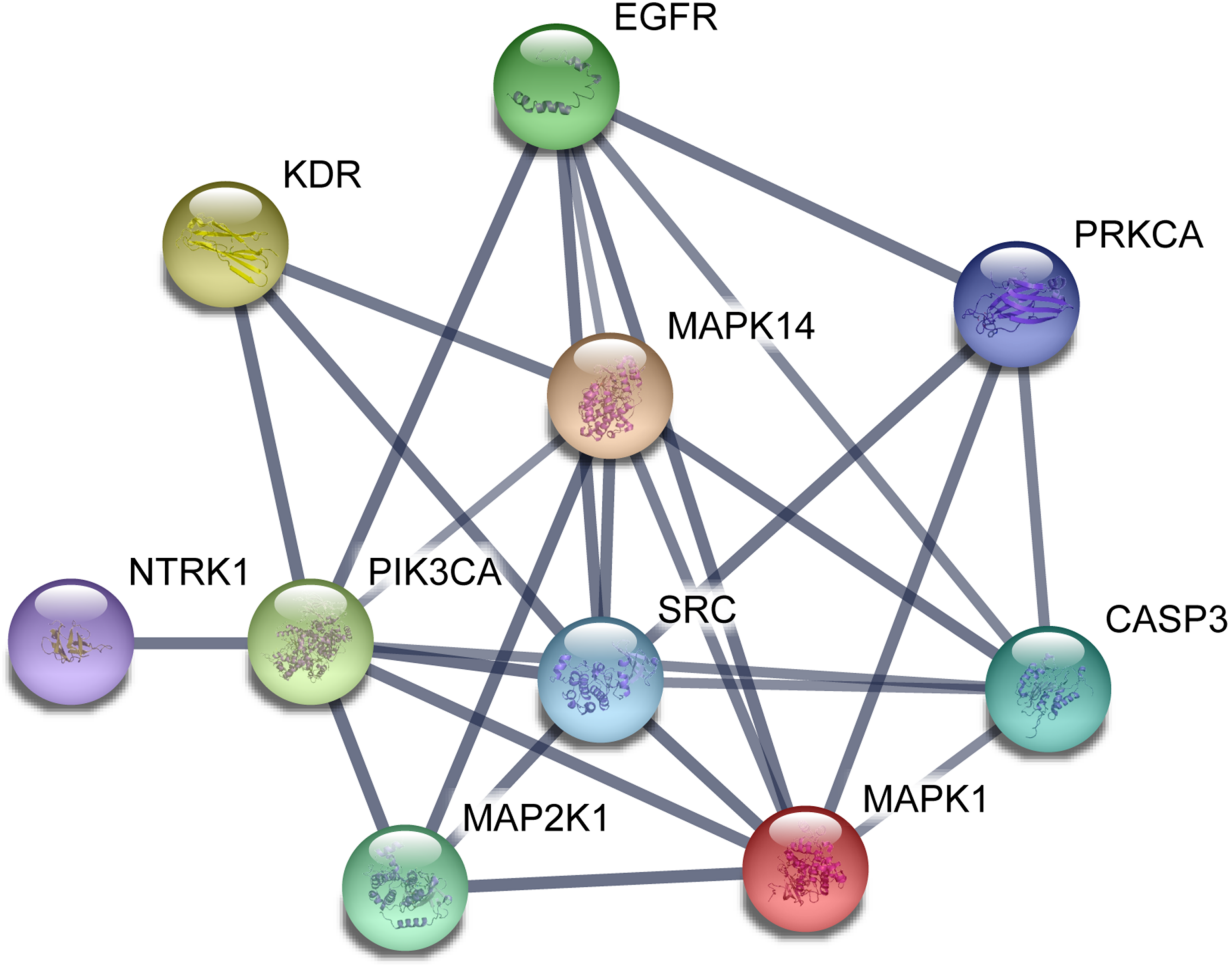
Interactions among the identified therapeutic targets for intraepidermal blistering induced by anti‐Dsg1/3 autoantibodies. The interaction map was drawn using STRING (string‐db.org; accessed 26 November 2018). The interactions are displayed in the confidence setting, where line thickness indicates the strength of data support at high confidence (0.7). The colour of nodes was selected at random. The following genes served as input: *EGFR* (EGFR), *PRKCA* (PKC), *SRC* (Src), *MAP2K1* (MEK), *KDR* (VEGFR2), *MAPK14* (p38 MAPK), *MAPK1* (ERK), *PIK3CA* (PI3Kα), *NTRK1* (TrkA), and *CASP3* (caspase‐3)

Given its rapid therapeutic effect, modulation of autoantibody‐induced intraepidermal blistering would be an ideal adjuvant treatment modality to corticosteroid/rituximab treatment because it would induce healing of lesions at a time at which corticosteroid/rituximab treatment is not yet effective. To address this unmet medical need and based on the notion of aberrant keratinocyte signalling in pemphigus (Pollmann et al., 2018), we used an unbiased screening approach to identify novel treatment targets for pemphigus that specifically modulate pemphigus autoantibody‐induced loss of keratinocyte adhesion. Through primary screening and multiple validation experiments, including an HSOC model, we identified four novel therapeutic targets that were able to modulate autoantibody‐induced intraepidermal blistering in pemphigus (Figure 7). Specifically, these targets are MEK1, TrkA, PI3Kα, and VEGFR2, as the corresponding inhibitors impaired anti‐Dsg1/3‐induced pathology in four different pemphigus model systems. In addition, we identified the p38 MAPK pathway, one of the best described anti‐Dsg3‐induced signalling cascades, as an important cause of pathology in the assays employed here. While pharmacological inhibition of the p38 MAPK pathway almost completely abolished the blister‐inducing activity of pemphigus patient IgG after injection into neonatal mice (Berkowitz et al., 2006), intraepidermal blistering was unaltered in epidermis‐specific p38α‐deficient mice (Mao et al., 2011), and pemphigus patients treated with p38 MAPK inhibitors experienced no clinical benefit (Sayar et al., 2014). Hence, p38 MAPK and its downstream signalling components, such as HSP27, are not likely to be therapeutic targets for pemphigus. Based on the results presented here and elsewhere (Hariton et al., 2017; Pollmann et al., 2018; Spindler & Waschke, 2018), EGFR (Sayar et al., 2014), VEGFR2, and TrkA, as well as their downstream targets (Figure 8), seem to be more promising therapeutic targets for modulating intraepidermal blistering induced by pemphigus autoantibodies. Modulation of these pathways may be achieved by either systemic or topical administration of compounds. As for corticosteroids (Joly et al., 2002), topical application should have significantly fewer adverse events than systemic treatment. Topical treatment using low MW compounds has already been described for a number of inflammatory skin diseases (Craiglow, Tavares, & King, 2016; Putterman & Castelo‐Soccio, 2018). Albeit for other, mostly oncological indications, compounds targeting the identified pathways are approved or are currently being evaluated in clinical trials, such as the VEGFR2 inhibitor apatinib (Miao et al., 2018), the TrkA kinase inhibitor CT327, which lessens itching in psoriasis patients as a topical formulation (Roblin et al., 2015), the PI3Kα/δ inhibitor copanlisib (Dreyling et al., 2017), and several MEK inhibitors (Sarkisian & Davar, 2018). Of these inhibitors, the topically applicable TrkA inhibitor CT327 seems the best suited for testing in pemphigus patients.

While we believe that screening using a complex biological system has several advantages over molecule‐based screening methods, the data obtained here should be interpreted within the limitations of the methodology. Due to the complexity of the Dsg3 internalization assay, only a limited number of experiments can be performed. The screening of 141 compounds with two replicates required 5–6 months of work by an experienced researcher. Furthermore, the complexity of the experiment induces variability. Of note, the results obtained with PV2 did not correlate with those obtained with PV10 (Pearson correlation). Hence, we decided to include any experiment at any concentration of potential drug candidates if the given compound had the predefined inhibitory effect on PV IgG‐induced Dsg3 internalization. Overall, this approach may introduce a bias towards not detecting true inhibitory compounds. When evaluating this possibility, the screening missed detecting EGFR (Sayar et al., 2014) because PD153035, a potent and specific EGFR inhibitor with an IC_50_ in the picomolar range (Fry et al., 1994), is present in the library. PKC (Seishima et al., 1999) was identified in the screening because enzastaurin impaired PV IgG‐induced Dsg3 internalization in both experiments. However, in the HaCaT cell dissociation assay, enzastaurin at 0.1 or 1.0 μM had no effect on PV IgG‐induced fragmentation, and at 10 μM, the compound increased cell fragmentation over twofold (*P* < 0.05, Kruskal–Wallis H test followed by Dunn's post hoc test). Caspase, ERK, and Src inhibitors were not present in the library.

We also did not differentiate between the differences in signalling events induced by anti‐Dsg1/3 and non‐Dsg antibodies (Walter et al., 2017). However, in most PV patients, autoantibodies to both Dsg1 and Dsg3 are present (Pollmann et al., 2018). Thus, screening with PV patient IgG rather than defined autoantibodies better reflects the situation encountered in patients. In addition, the use of HaCaT cells, an immortalized cell line with potentially different signalling pathways compared to non‐immortalized cells, may have contributed to missing some compounds that would have inhibitory effects in other cell culture systems. Hence, taken together, our screening approach was biased towards false negative results.

We decided to use the anti‐Dsg1/3 scFv in the HSOC model as opposed to pemphigus mouse models in neonatal mice or IgG preparations from pemphigus patients, because the neonatal mouse skin is quite different from human adult skin, and patient IgG does not consistently induce splitting in the skin organ culture model (not shown). The ex vivo model used in this study is well‐standardized. While, due to relatively low variability, this is an advantage in identifying potential new pathways in pemphigus, the model has to be interpreted within its limitations. Specifically, while we detect scFv binding in the model, the protein expression (determined by immunohistochemistry) of Dsg1/3 is not grossly altered (not shown). Hence, the precise mechanisms by which blistering occurs in this model, as well as in patients, need to be addressed in more detail in further experiments (Sajda & Sinha, 2018).

The advantage of using a complex system as opposed to an enzymic assay for a known target is the fact that no therapeutic target has to be known a priori. Hence, screening in biological systems can be performed in an unbiased fashion without prior insights into the molecular mechanisms of the disease. In addition, if a desired (inhibitory) effect is observed, the inhibited pathway is likely of functional relevance.

In summary, we provide novel insights into PV IgG‐induced signalling events in keratinocytes, which contribute to skin pathology in pemphigus. Furthermore, by applying an unbiased screening approach, with subsequent validation in a standardized skin organ culture model, we were able to identify four novel potential therapeutic targets for the treatment of pemphigus.

## AUTHOR CONTRIBUTIONS

I.A.K.B., S.F., C.S.T., A.K., N.F., M.K., K.K., V.B.B., and J.E.H. performed the experiments; E.S. and N.v.B. sampled and characterized the patient material; C.M.H. and S.E. produced and purified the anti‐Dsg1/3 scFv; C.M.H., S.E., and J.E.H. established the human skin organ culture model; and R.J.L., J.E.H., and D.Z. concepted the study. All authors wrote and revised the manuscript.

## CONFLICT OF INTEREST

R.J.L. has received research funding from Miltenyi Biotec, Biogen, Biotest, Almirall, True North Therapeutics, UCB Pharma, ArgenX, TxCell, Topadur, Incyte, and Admirx and fees for consulting or speaking from ArgenX, Immunogenetics, Novartis, and Lilly. D.Z. has received support through research and development grants as well as for consulting or lecturing from Biotest, Fresenius, Miltenyi Biotec, Roche Pharma, Biogen, AbbVie, UCB, Janssen, Euroimmun, Dompe, Novartis, and ArgenX. C.M.H. is advisor to ArgenX and viDa Therapeutics. All other authors declare no conflicts of interest.

## DECLARATION OF TRANSPARENCY AND SCIENTIFIC RIGOUR

This Declaration acknowledges that this paper adheres to the principles for transparent reporting and scientific rigour of preclinical research as stated in the *BJP* guidelines for Design and Analysis, and Immunoblotting and Immunochemistry, and as recommended by funding agencies, publishers and other organizations engaged with supporting research.

## Supporting information


**Table S1.** Supporting Information
